# Human Immunodeficiency Virus Infection and Pregnancy

**DOI:** 10.1155/S1064744994000438

**Published:** 1994

**Authors:** Richard H. Schwarz

**Affiliations:** Clinical Affairs State University of New York Health Science Center at Brooklyn 450 Clarkson Avenue, Box 12 Brooklyn NY 11203 USA

## Abstract

The human immunodeficiency virus (HIV) epidemic is clearly one of the most serious health-care crises in the professional lives of contemporary physicians. It cannot be regarded as a curiosity to be dealt with by inner-city infectious-disease experts, but rather must be considered a problem for all health-care providers and a problem in which the obstetrician-gynecologist has a special role to play.


Infectious Diseases in Obstetrics and Gynecology 2:76–82 (1994)
© 1994 Wiley-Liss, Inc.

### Human Immunodeficiency Virus Infection and Pregnancy

**Richard H. Schwarz**
*Clinical Affairs, State University of New York, Health Science Center at Brooklyn, Brooklyn, NY*

ABSTRACT

**The human immunodeficiency virus (HIV) epidemic is clearly one of the most serious health-care crises in the professional lives of contemporary physicians. It cannot be regarded as a curiosity to be dealt with by inner-city infectious-disease experts, but rather must be considered a problem for all health-care providers and a problem in which the obstetrician-gynecologist has a special role to play.** © 1994 Wiley-Liss, Inc.

KEY WORDS
AIDS, perinatal transmission, AZT, pneumocystis

Has the human immunodeficiency virus (HIV) epidemic really abated? There is evidence of a leveling off of new cases in the gay male population generally, although a recent resurgence has been reported among young gay males in San Francisco. There is no indication of a decrease in new cases in drug abusers, their sexual partners, or their children. In many of America's inner cities, acquired immunodeficiency syndrome (AIDS) is the leading cause of death among women in the reproductive-age group and a more frequent cause of perinatal death than any of the so-called "TORCH" infections. AIDS in drug users is a disease of families, leaving growing numbers of youth orphaned. An estimation has been made that, unless the course of the epidemic changes, 80,000 children and adolescents will be orphaned by the year 2000.[1] Even in areas of lower prevalence, it behooves the obstetrician to consider the diagnosis and offer testing along with appropriate counseling to his or her patients.

### EPIDEMIOLOGY

As of September 30, 1993, 339,250 cases that meet the Centers for Disease Control (CDC) definition of AIDS have been reported in the United States. Newly expanded definitions will increase the accumulation rate. New York City, NY, has reported 56,581 cases, accounting for 17% of the national total. Of these, 18% are women and 2% are children.[2] As many as 200,000 individuals (3.5%) of the population of that city are estimated to be HIV positive. This means that, for every symptomatic individual, 20–30 individuals have no symptoms at all and are, for the most part, unaware of their infection. Fewer than 5% of those infected can be expected to develop AIDS within 5 years, and the mean interval is more than 10 years. Available prospective data suggest that, barring dramatic therapeutic developments, 99% of infected individuals will probably go on to develop symptoms.[3] Although there are no good data concerning women, the median survival in men following the diagnosis is 24 months with a 5-year survival of 3.4%. This rate will undoubtedly increase as more effective therapy is applied. The burden on our health-care system is enormous. On any given day in New York City, more than 3,000 acute-care beds are occupied by patients with HIV infection.

Drug use now accounts for the majority of new cases of HIV infection and nearly all perinatal HIV infections. Of the estimated 200,000 intravenous (IV)-drug users in New York, of whom 75% are

Address correspondence/reprint requests to Dr. Richard H. Schwarz, State University of New York, Health Science Center at Brooklyn, 450 Clarkson Avenue, Box 12, Brooklyn, NY 11203.

Received February 18, 1994
Accepted June 15, 1994
Clinical Study males, most are sexually active and 50–70% are HIV positive. Drug-use patterns change, and there has been a recent resurgence of heroin use along with increased polydrug abuse. Crack cocaine has also contributed to the problem as its use has increased. Because it is rapidly addicting and short acting, needs are great and often met by women users through the exchange of sex-for-drugs. This situation has led to a concomitant dramatic increase in sexually transmitted diseases along with a failure to obtain adequate or often any prenatal care. The Kings County Hospital in Brooklyn sees significant numbers of patients who have received no prenatal care. In a survey of unregistered patients, 48% were found to have positive urine screens for cocaine metabolites; 17% had positive serologies for syphilis, a rate 1,400 times the national average; and 7% were HIV positive.

Because of the association between poverty and drug abuse in inner-city populations as well as of secondary heterosexual spread, HIV infection disproportionately affects ethnic minorities. In New York, 63% of males with HIV are black or Latino, while the figure for women is 85% and for children 90%.[2]

Both the absolute numbers and the percentage of women with HIV infection are increasing. Among women with symptomatic AIDS, 61% of the cumulative cases are the result of their own drug use, while 23% of cases are assigned to drug-using sexual partners.[2] AIDS is the leading cause of death in women between ages 15 and 44 years in New York and the fifth leading cause of death of all American women in the reproductive-age group. Women, more often than men, have no identifiable risk behavior which poses a significant problem when selective screening is employed. The seroprevalence in obstetric populations varies widely from 0.15% in the United States to a high of 12% in Zambia in Central Africa. A statewide anonymous cord-blood survey in New York showed a prevalence of 1/61 (1.6%).[4]

### IMPACT OF PREGNANCY ON THE COURSE OF HIV INFECTION

The concern is whether or not the altered immune status of pregnancy might increase the morbidity or mortality from HIV infection as it is thought to do with other viral diseases such as influenza, hepatitis, and varicella, in which the host response is largely cell mediated.[5] Early reports included case studies of mortalities in symptomatic pregnant women. In fact, the first 5 patients reported with AIDS in pregnancy all died of opportunistic infections.[6–8] Such mortality data must be viewed cautiously, however, since they may reflect reporting bias, or delay, in diagnosis and treatment during pregnancy, perhaps because of fetal concerns. Another perspective can be obtained from the follow-up of asymptomatic women whose seropositivity is detected by their having delivered children who developed AIDS. These women have generally done poorly, with the majority progressing to symptomatic disease in a relatively short period of follow-up.[9] An alternative explanation is that these women were simply prone to both perinatal transmission and disease progression. Prospective studies comparing HIV-positive pregnant and nonpregnant women are ongoing, and the available reports demonstrate little or no difference in the rate of clinical or immunologic deterioration.[10,11] It is likely that a more important determinant is the stage of the HIV infection at the time of the pregnancy. In a study in which HIV-positive gravidas were monitored with CD4 counts, those with counts <300 proved more likely to develop opportunistic infections during pregnancy,[12] raising the question of the use of prophylactic azidothymidine (AZT) or pentamidine under such circumstances.

### IMPACT OF HIV INFECTION ON PREGNANCY OUTCOME

Most studies fail to demonstrate an impact of asymptomatic HIV infection on the common parameters of pregnancy outcome such as birth weight, preterm delivery, and premature rupture of the membranes.[13–15] Reports that seem to show an effect on outcome include more women with symptoms, indicating a more advanced stage of HIV infection.[9,16] These studies are also complicated by confounding variables such as poor nutrition and substance abuse, making them even more difficult to interpret.

The earliest reports of the rate of perinatal transmission varied widely, and prospective studies were impeded by the lack of a practical antigen test and the fact that passively acquired antibody in the newborn may take as long as 15 months to clear. Other methods, such as polymerase chain reaction, viral culture, and IgA antibody determination, are being explored to facilitate earlier neonatal diagnosis.[17] The best estimate of the rate of vertical transmission is 20–30% with one notable exception. The European collaborative study reported a rate of 13%.[18] With a view toward developing a means of fetal protection, we have focused considerable attention on the factors that might influence the rate of perinatal transmission. An advanced stage of disease and CD4 counts of $<400/mm^3$ are associated with an increased rate of transmission. While a few studies have suggested that maternal antibodies to certain epitopes of the GP120 envelope glycoprotein seem to reduce the rate,[19] other studies have not been able to replicate the latter findings.[20] Considerable attention is now focused on the timing and mechanisms of maternal-fetal transmission, but these factors are not yet thoroughly clarified. HIV can be transmitted as either free virus or cell-associated virus, in this case, in mononuclear cells. Studies have demonstrated that maternal mononuclear cells can enter the fetal circulation and can be identified in 10–20% of pregnant women. HIV can also be recovered from cord blood in 20–30% of infected women and has been isolated from aborted central nervous system (CNS) tissue at 20 weeks gestation.[21] Both free and cell-associated viruses have been isolated by cervical and vaginal lavage, making intrapartum transmission a possibility.[22] Although prior studies have indicated that a cesarean section does not prevent perinatal infection,[18] more recent evidence indicates that it may be at least partially protective. Trials are also underway to determine whether AZT administration antepartum, intrapartum, or during the neonatal period can reduce transmission.

As concerns the neonatal outcome, 93% of the pediatric cases in New York are the result of perinatal transmission and 78% of those cases relate to drug use.[2] Of the remaining cases, blood transfusion accounts for most, but there is insufficient information to assign a category in 3% of the cases. Significantly, in only 11% of the perinatally transmitted cases did the parents have diagnosed AIDS at the time the diagnosis was established in the child. The prognosis in these children is often poor despite aggressive treatment. There do, however, appear to be 2 groups of children with HIV infection with different clinical courses. One group includes those who develop symptoms in the first 6 months of life and rarely survive beyond 1 year of age. Increasingly, we are recognizing a second group that includes those who remain mildly infected for several years. Characteristically, those in the latter group have interstitial pneumonitis while those with the acute short course are more likely to have pneumocystis pneumonia or encephalitis.[23]

### ROLE OF THE OBSTETRICIAN

### Education and Testing

The first challenge and the primary role for the obstetrician lie in counseling and testing since, in most circumstances, once an infected patient is identified, she will be referred to or at least comanaged with an expert in HIV. From the onset of the epidemic, questions have been raised in determining who should be tested over the conflicting principles of public health vs. the protection of an individual's civil rights. Some have pressed for mandatory testing, citing historic examples such as syphilis. That approach poses several problems. One concern is that there have been, and continue to be, violations of the civil liberties of families and individuals which are not prevented by our current laws.[24–26] Another concern is that mandatory testing may cause women who might be at risk to avoid care for fear of being identified. Perhaps the greatest concern, at least at the outset of the epidemic, and the major flaw in the syphilis analogy, is that there is so little to offer from a therapeutic or preventive standpoint.[25] This argument has become less compelling as more effective therapy has become available. Mandatory testing also fails to provide the protection providers seek for themselves, since the screening detects antibodies that, in the extreme case, may not appear for 18 months after infection. The availability of a reliable antigen test could obviate this problem.

Another option posed was premarital testing, but it too has serious problems. The results in Illinois were disastrous with exorbitant costs per case identified and a marked drop in marriage licenses issued, indicating that couples likely obtained their licenses in neighboring states. Currently, the most common approach is testing based upon the identification of patients with risk factors such as immigration from an endemic area like Central Africa, a bisexual partner, or a history of transfusion prior to the institution of screening the blood supply. The difficulty with selective testing, especially in high-prevalence areas, is that a significant number of seropositive individuals will have no history of risk factors.[27] In one study, more than 40% of prenatal patients who subsequently proved to be seropositive gave no history of risk factors when questioned by specially trained interviewers. Health departments often report lower figures for the absence of risk factors, but this information is generally obtained at interviews with the test results in hand.

Because of these data and because the seroprevalence in the obstetric population at our institution exceeds 2% and that of the State of New York is 1.6%,[4] we have elected to counsel and offer testing to all pregnant women. If risk factors are present, the counseling is more positive. This policy of "informed refusal" permits women to reject testing after being appropriately counseled. This approach has now been adopted by the New York State Department of Health. The downside is the burden of providing proper counseling to a large population of patients, since it is both time and personnel intensive and cannot be done effectively in groups. It also requires educating the counselors, be they obstetricians, midwives, nurses, or other health-care professionals, in order to ensure the quality of the information in providing a proper basis for patients' decisions. The most recent guidelines from the CDC urge us to recommend, offer, or make available testing, depending upon the local prevalence. It should be noted that the false-positive rate is very high with enzyme-linked immunosorbent assay (ELISA) screening alone, especially in low-prevalence populations.[28] These results must be confirmed by a more specific test such as the Western blot.

When this "informed refusal" approach was initiated, 40–50% of our patients accepted testing and 3% were positive, a prevalence slightly higher than the overall rate, perhaps indicating a degree of self-selection. When the counseling language was changed from "offering" to "recommending" testing, the acceptance rate rose to 70%. It is also important to note that, in follow-up counseling, a significant number of women will continue to deny risk behavior and only a small number, approximately 10%, will opt for pregnancy termination when identified sufficiently early to permit it.[29] There is still no universally accepted approach to testing; however, as more effective therapy becomes available, it will be increasingly acceptable to recommend testing to all pregnant women.[25]

Testing of physicians and other health-care personnel is also a controversial issue. While there was considerable notoriety concerning a Florida dentist who allegedly infected several of his patients, the facts in that case remain less than clear. Although physician-to-patient transmission is theoretically possible, its frequency is exquisitely low and thoroughly documented cases are rarely found in the literature. Fortunately, an effort by the CDC to develop a list of "risk-prone procedures" that could not be performed by HIV-positive physicians was thwarted for lack of any scientific basis. The current policy specifies that the clinical activities of an HIV-positive physician should be determined and monitored locally by a committee of doctors expert in the area, including the physician's own care providers.

### Counseling

Although some patients are aware of their HIV status at the time they seek prenatal care, a more common scenario is that their seropositivity is discovered as a result of the initial counseling and testing at the first prenatal visit. The next step is post-test counseling. This step is also important for the woman who has a negative serology, especially if she continues her risk behavior despite the physician's advice.[26] Periodic testing should be done at an interval sufficient to allow for seroconversion. The standard recommendation is 6 months although the conversion interval may vary from the usual 6–12 weeks to as long as 18 months.

For the HIV-positive woman, counseling should address the following issues:[26,30]

1. A description of the early manifestations of AIDS and the need to seek attention when they occur.
2. An accurate understanding of the prognosis, which is obviously difficult considering its nature.
3. The need to practice safe sex and avoid needle sharing.
4. A prohibition from donating blood or organs.
5. Advice against sharing implements that might

**TABLE 1. Protocol for HIV-positive pregnant patients[38]**

| |
|---|
| Prenatal |
| CD4 count each trimester (more frequently if low) |
| Tests at first visit |
| Toxoplasmosis antibody |
| Cytomegalovirus antibody |
| Mantoux tuberculin test |
| Hepatitis surface antigen |
| B-2 macroglobulin |
| VDRL |
| Gonococcal culture |
| Chlamydia culture |
| Cryptococcal antigen |
| SMA-12 |
| Pneumovax influenza vaccine and hepatitis B vaccine administered as indicated |
| Tests at delivery |
| Urinalysis for cytomegalovirus |
| Repeat toxoplasmosis antibody and cytomegalovirus antibody if negative at first visit |

be contaminated with blood, such as razors or toothbrushes.

6. Advice that the test results should be shared with sex and needle-sharing partners and that they should seek counseling and testing as well.
7. Advice that health-care workers dealing with the patient should be informed by the patient of her HIV positivity. Unfortunately, this information often makes it difficult for the patient to obtain care. The medical record is the ideal place to communicate with other health professionals, but access to these records is often not limited to those with legitimate need.

### Antenatal Management

Counseling and psychosocial support form the cornerstone of antenatal management. These steps must be taken, if possible, sufficiently early in pregnancy to empower all of the patient's reproductive options. Whether or not the patient chooses to continue the pregnancy, the stresses are enormous and support systems must be developed and maintained throughout and beyond the pregnancy.

Screening for other infections, such as toxoplasmosis and cytomegalovirus, is critical since sexual as well as perinatal transmission are common to a number of these diseases. The procedures to be carried out prenatally are listed in Table 1. The patient should receive pneumococcal influenza vaccine (Pneumovax, Merck, Sharp & Dohme, West Point, PA) and, if indicated by a negative surface-antigen test, hepatitis B vaccine. The regular measurements of CD4 counts can identify patients at risk for disease progression and opportunistic infection. If the count falls below 500, the use of zidovudine (Retrovir AZT, Burroughs Wellcome Co., Research Triangle Park, NC) should be discussed with the patient. While almost all would recommend AZT if the count falls to 200, the 200–500 range remains a debatable area.[31,32] AZT has not been conclusively evaluated in pregnancy; however, animal studies have not demonstrated either mutagenicity or teratogenicity. AZT acts by inhibiting reverse transcriptase, and this mechanism of itself poses no specific threat. AZT does, however, cross the primate placenta and, consequently, has the potential to cause anemia in the fetus as it does in the adult.[33] In addition to monitoring the maternal hemoglobin, it is well to monitor the fetus with ultrasound for growth retardation or the possibility of fetal hydrops which could result from severe fetal anemia. Recent reports suggest that the anemia in adults can be reversed by the use of erythropoietin. More information is being gathered on AZT in pregnancy, but, for now, we must maintain a balance in our thinking in order not to place the mother at risk because of fetal concerns. It is not prudent to recommend routine AZT for all HIV-positive pregnant women, but it should be considered for those with low CD4 counts. Although we have no data concerning women, clearly, a count <200 indicates a significant risk for pneumocystis.[34] For this reason, a woman with a count <200 should be offered aerosol pentamidine prophylaxis. There are no conclusive studies on the safety of pentamidine during pregnancy; however, when it is administered by aerosol, serum concentrations reach only 5% of the dose given. Trimethoprim/sulfamethoxazole, now the first line, is an effective alternative approach. Its side effects include fever, rash, and neutropenia.[35]

It may be more difficult to evaluate the HIV-infected patient during pregnancy because some symptoms such as fatigue and weight loss might be dismissed as pregnancy related when, in fact, they indicate a progression of HIV disease. Patients should be advised to report all symptoms. They also should be provided with nutritional counseling and encouraged to maintain normal weight gain.

Should an opportunistic infection develop, consultation with an infectious-disease specialist is essential since these infections are complex to manage and life threatening. Generally, there is little reason to withhold any antibiotic in the face of such a grave prognosis. The most common opportunistic infection, particularly in pregnancy, is *Pneumocystis carinii* pneumonia and the accepted treatment is trimethoprim/sulfamethoxazole along with pentamidine. Concerns about sulfa competing for bilirubin-binding sites in the fetus are overstated, especially under these circumstances. Other opportunistic infections that occur much less frequently in pregnancy include CNS toxoplasmosis; oral, vaginal, and esophageal candidiasis; cryptococcus meningitis; cytomegalovirus; and herpes. Recently, drug-resistant tuberculosis has emerged as an increasingly frequent and difficult problem.

### Labor Management

Although no controlled studies back this recommendation, the use of scalp sampling and scalp electrodes is not advisable since the best protection for the fetus is intact skin.[30] The current recommendation as to the mode of delivery is that a cesarean section be reserved for obstetric indications only and that this procedure does not prevent perinatal transmission. In an earlier German study in which elective cesarean delivery was carried out in consecutive HIV-positive women, 9/20 infants were infected despite abdominal delivery. More recently, studies of twins suggested the highest risk for a first-born twin delivered vaginally; a lower risk for a first-born twin delivered by cesarean section; and the lowest risk for a second-born twin.[36] The implications are that further quantitatively important studies could well lead to revised policies regarding the mode of delivery. The newborn should be carefully cleansed of maternal blood and secretions, with particular attention to preparation of the infant's skin before drawing blood.

### Postpartum Management

There is little information about the postpartum course in HIV-infected women, but no evidence that it is significantly altered. The virus has, however, been isolated from breast milk and case reports indicate that mothers have acquired HIV infection from postpartum transfusions and then transmitted it to their infants by breast-feeding. In another report in which known seropositive women breast-fed their infants, the rate of vertical transmission was no greater than that of women who did not breast-feed.[37] Breast-feeding should be avoided if there is a suitable alternative, as there is in the developed world. Obviously, in some parts of the world, this alternative does not exist. A final step in the care of the infected pregnant woman is to make certain that she receives continuing care from a knowledgeable and sensitive physician, which is not always a simple matter because not a large number of physicians are willing to provide such care.

### CONCLUSIONS

The HIV epidemic is clearly one of the most, if not the most, serious health-care crises in the professional lives of contemporary physicians. It cannot be regarded as a curiosity to be dealt with by innercity infectious-disease experts, but rather must be considered a problem for all health-care providers and a problem in which the obstetrician-gynecologist has a special role to play.

### REFERENCES

1. Michaels D, Lavine C: Estimates of the number of motherless youth orphaned by AIDS in the United States. JAMA 268:3456–3461, 1992.
2. New York City Department of Health: AIDS Surveillance Update. October 1993.
3. Centers for Disease Control: First 100,000 cases of AIDS, United States. MMWR 38:561–563, 1989.
4. Novick LF, Berus D, Stoicol R: HIV seroprevalence in newborns in New York State. JAMA 261:1745–1750, 1989.
5. Nanda D, Minkoff HL: HIV in pregnancy-transmission and immune effects. Clin Obstet Gynecol 32:456–466, 1989.
6. Jensen LP, O'Sullivan MJ, Gomez del Rio M, Acquired immunodeficiency syndrome in pregnancy. Am J Obstet Gynecol 148:1145, 1984.
7. Minkoff HL, DeRegt RH, Landesman S, Schwarz RH: *Pneumocystis carinii* pneumonia associated with acquired immunodeficiency syndrome in pregnancy: A report of three maternal deaths. Obstet Gynecol 67:284, 1986.
8. Wetti CV, Roldan EO, Fujaco RM: Listeriosis as a cause of maternal death: An obstetric complication of acquired immunodeficiency syndrome. Am J Obstet Gynecol 147:7, 1983.
9. Minkoff H, Nanda D, Menez R, Fikrig A: Pregnancies resulting in infants with acquired immunodeficiency syndrome or AIDS related complex. Obstet Gynecol 69:285, 1987.
10. Bigger RJ, Palma S, Landesman S, Goedert JJ: Helper and suppressor lymphocyte changes in HIV infected mothers and their infants. Abstract No. 4032. Fourth International Conference on AIDS, Stockholm, 1988.

11. Schaefer A, Grosch-Woerner I, Friedman W, Kunzer R, Melke M, Jimenez E: The effect of pregnancy on the maternal course of HIV disease. Abstract No. 4039. Fourth International Conference on AIDS, Stockholm, 1988.
12. Minkoff HL, Willoughby A, Mendez H: Serious infection during pregnancy among women with advanced immunodeficiency virus infection. Am J Obstet Gynecol 162:30, 1990.
13. Johnstone FD: Management of pregnancy in women with HIV infection. Br J Hosp Med 48:664–670, 1992.
14. Johnstone FD, McCallum L, Brettle R, Ingles JM, Pentherer H: Does HIV infection affect the outcome of pregnancy? Br Med J 296–467, 1988.
15. Semperimi AE, Vacetich A, Pardi G, Cossu MM: HIV infection and AIDS in newborn babies of mothers positive for HIV antibodies. Br Med J 294:610, 1987.
16. Iosub S, Bonji M, Stone RK: More on HIV embryopathy. Pediatrics 80:512, 1987.
17. Schafer A, Koch MA: Risk factors and diagnostic tools for the detection of maternal fetal HIV-transmission. J Prenat Med 19(Suppl 1):252–256, 1991.
18. European Collaborative Study: Children born with HIV-1 infection: Maternal history and risk of transmission. Lancet 337:253–259, 1991.
19. Ryder RW, Nsa W, Hassg SE, et al.: Prenatal transmission of human immunodeficiency virus type 1 to infants of seropositive women in Zair. N Engl J Med 330:1037–1042, 1989.
20. Goedert JJ, Drummond JE, Minkoff HL, et al.: Mother-to-infant transmission of human immunodeficiency virus type 1 associated with prematurity or low anti-gp120. Lancet 11:1351–1354, 1989.
21. Joviasas E, Koch MA, Sdiafer A, Stanber M, Lowenthal D: LAV/HIV-III in 20 week fetus. Lancet 2:29, 1985.
22. Pomerantz RJ, dala Monte SM, Donegan SP, et al.: Human immunodeficiency virus (HIV) infection of the uterine cervix. Ann Intern Med 108:321–327, 1988.
23. Kilbey MM, Klusheed A: Methodological issues in epidemiological prevention and treatment research on drug-exposed women and their children. NIDA Res Monogr 117:166–182, 1992.
24. Blendon RJ, Donelan K: Discrimination against people with AIDS: The public's perspective. N Engl J Med 319:1022–1026, 1988.
25. Moreno JD, Minkoff HL: Human immunodeficiency virus infection during pregnancy. Clin Obstet Gynecol 35:813–820, 1992.
26. Tuomala R: Human immunodeficiency virus education and screening of prenatal patients. Obstet Gynecol Clin North Am 17:571–583, 1990.
27. Minkoff HL, Landesman SH: The case for routinely offering prenatal testing for human immunodeficiency virus. Am J Obstet Gynecol 159:793–796, 1988.
28. Sherer R: Physician use of the HIV antibody list: The need for consent, counselling, confidentiality and caution. JAMA 259:264–265, 1988.
29. Sehwyn PA, Carter RJ, Schoemlaur EE: Knowledge of HIV antibody status and decisions to continue or terminate pregnancy among intravenous drug users. JAMA 261:356–371, 1989.
30. Minkoff HL: Care of pregnant women infected with human immunodeficiency virus. JAMA 258:2714–2717, 1987.
31. Fischl MA, Richman DD, Greco MH: The efficacy of azidothymidine (AZT) in the treatment of patients with AIDS and AIDS related complex. A double-blind, placebo controlled trial. N Engl J Med 317:185–191, 1987.
32. Volberding PA, Lagakos SW, Koch MA: Zidovudine in asymptomatic human immunodeficiency virus infection: A controlled trial in persons with fewer than 500 CD4 positive cells per cubic millimeter. N Engl J Med 322: 941–949, 1990.
33. Watts DH, Brown ZA, Tartaghone T: Pharmacolinetic disposition of zidovudine during pregnancy. J Infect Dis 163:226–232, 1991.
34. Phair J, Muno A, Detels R, Kaslow R, Rinaldo C, Saah A: The risk of *Pneumocystis carinii* pneumonia among men with trauma immunodeficiency virus type 1. N Engl J Med 322:1607–1608, 1990.
35. Fischl MA, Dickinson GM, LaVoce L: Safety and efficacy of sulfamethoxazole and trimethoprim prophylaxis for *Pneumocystis carinii* pneumonia in AIDS. JAMA 259: 1185–1189, 1988.
36. Goedert JJ, Duleige AM, Amos CI, Felton S, Biggar RJ: High risk of HIV-1 infection for first born twins. Lancet 338:1471–1475, 1991.
37. VandePerre P, Papage P, Homsey J, Dabis F: Mother-to-infant transmission of human immunodeficiency virus by breast milk: Presumed innocent or presumed guilty. Clin Infect Dis 15:502–507, 1992.
38. Nanda D: Management of HIV in pregnancy. Obstet Gynecol Clin North Am 17:617–623, 1990.
